# Lightweight cotton diseases real-time detection model for resource-constrained devices in natural environments

**DOI:** 10.3389/fpls.2024.1383863

**Published:** 2024-06-06

**Authors:** Pan Pan, Mingyue Shao, Peitong He, Lin Hu, Sijian Zhao, Longyu Huang, Guomin Zhou, Jianhua Zhang

**Affiliations:** ^1^ Agricultural Information Institute, Chinese Academy of Agricultural Sciences, Beijing, China; ^2^ National Agriculture Science Data Center, Beijing, China; ^3^ National Nanfan Research Institute (Sanya), Chinese Academy of Agricultural Sciences, Sanya, China; ^4^ Institute of Cotton Research, Chinese Academy of Agricultural Sciences, Anyang, China; ^5^ Farmland Irrigation Research Institute, Chinese Academy of Agricultural Sciences, Xinxiang, China

**Keywords:** cotton diseases detection, natural environment, deep learning, lightweight, YOLOv8

## Abstract

Cotton, a vital textile raw material, is intricately linked to people’s livelihoods. Throughout the cotton cultivation process, various diseases threaten cotton crops, significantly impacting both cotton quality and yield. Deep learning has emerged as a crucial tool for detecting these diseases. However, deep learning models with high accuracy often come with redundant parameters, making them challenging to deploy on resource-constrained devices. Existing detection models struggle to strike the right balance between accuracy and speed, limiting their utility in this context. This study introduces the CDDLite-YOLO model, an innovation based on the YOLOv8 model, designed for detecting cotton diseases in natural field conditions. The C2f-Faster module replaces the Bottleneck structure in the C2f module within the backbone network, using partial convolution. The neck network adopts Slim-neck structure by replacing the C2f module with the GSConv and VoVGSCSP modules, based on GSConv. In the head, we introduce the MPDIoU loss function, addressing limitations in existing loss functions. Additionally, we designed the PCDetect detection head, integrating the PCD module and replacing some CBS modules with PCDetect. Our experimental results demonstrate the effectiveness of the CDDLite-YOLO model, achieving a remarkable mean average precision (mAP) of 90.6%. With a mere 1.8M parameters, 3.6G FLOPS, and a rapid detection speed of 222.22 FPS, it outperforms other models, showcasing its superiority. It successfully strikes a harmonious balance between detection speed, accuracy, and model size, positioning it as a promising candidate for deployment on an embedded GPU chip without sacrificing performance. Our model serves as a pivotal technical advancement, facilitating timely cotton disease detection and providing valuable insights for the design of detection models for agricultural inspection robots and other resource-constrained agricultural devices.

## 1 Introduction

Cotton, a member of the Malvaceae family (Chohan et al., 2020), holds the top position among natural fibers, thanks to its simplicity of cultivation and its wide range of uses in clothing and home textiles. It satisfies nearly 35% of the global annual fiber demand (Huang et al., 2021). Beyond the textile industry, cotton plays a crucial role in the production of animal feed and edible oil (Townsend, 2020; Zaidi et al., 2020). In 75 countries, cotton crop production supports the livelihoods of over 250 million people (Wang et al., 2020).

Throughout the cotton growth cycle, diseases can significantly hinder both yield and quality, posing a substantial threat to the economic viability of farmers (Chi et al., 2021). According to statistics, estimates of the total cotton disease losses ranged from 6% to 12% of the yield lost due to disease (Lawrence et al., 2022). Among cotton diseases, verticillium wilt (Cai et al., 2009), fusarium wilt (Wang et al., 2009), and anthracnose (Nawaz et al., 2018) are particularly significant (Toscano-Miranda et al., 2022). They are often referred to as the ‘cancer’ of cotton crops due to their ability to substantially reduce cotton production.

The battle against cotton diseases endures, with ongoing efforts to avert crop losses by early and effective disease detection, followed by timely intervention (Mohanty et al., 2016; Guo et al., 2022). While manual disease detection is the prevailing approach, it is hampered by reliability issues and is impractical for large-scale monitoring due to time and cost constraints (Peyal et al., 2022). The quest for automated cotton disease detection methods is becoming increasingly urgent, particularly given the rapid growth of the cotton industry (Pan et al., 2023b).

Over the past two decades, image-processing techniques for identifying plant diseases have yielded promising results (Thakur et al., 2022). With recent advancements in machine learning, these techniques offer the potential to reduce labor costs, minimize time wastage, and enhance plant quality (Wani et al., 2022). However, traditional machine learning algorithms predominantly rely on manually crafted, low-level visual features based on engineering experience. This limitation often leads to subpar performance when dealing with complex scenes (Wang et al., 2022b). Consequently, further research is required to develop more efficient and automated methods (Zhang et al., 2023e).

Deep learning algorithms exhibit the capability to autonomously extract and learn complex high-level features through deeply structured convolutional neural networks. Due to its rapid evolution, deep learning models have been constructed for the detection of plant diseases (Pan et al., 2023a). These models not only excel in disease classification but also accurately determine disease locations on plant leaves within images (Liu and Wang, 2021). Much like other research domains such as medical science, mechanical automation, and logistics, the integration of robotics and deep learning into agriculture has sparked a revolution in the way plants are cultivated and safeguarded (Balaska et al., 2023). This transformative approach allows for the intelligent application of chemical sprays, including fungicides, herbicides, and pesticides, following successful robotic disease detection. This intelligent strategy offers the promise of establishing a cost-effective crop protection system (Saleem et al., 2021). This innovative approach has been applied to a wide range of crops, including cucumber (Li et al., 2023b), maize (Leng et al., 2023), potato (Johnson et al., 2021; Dai et al., 2022), rice (Jia et al., 2023), soybeans (Zhang et al., 2021), strawberry (Zhao et al., 2022), tomato (Tang et al., 2023b), and wheat (Zhang et al., 2023a), on a global scale for disease detection using deep learning techniques.

In recent years, researchers have harnessed deep learning techniques for the detection of cotton diseases. Several noteworthy studies have been conducted: Susa et al (Susa et al., 2022). applied the YOLOv3 algorithm to detect and classify cotton plants and leaves, achieving a remarkable mean Average Precision (mAP) score of 95.09%. Zhang et al (Zhang et al., 2023b). optimized the YOLOv5 algorithm to address the issue of subpar small target detection in the context of cotton wilt disease. They introduced a small target detection layer and incorporated an attention mechanism, resulting in an impressive mAP score of 91.13%. PRIYA et al (Priya et al., 2021). utilized Faster R-CNN with Region Proposal Network (RPN) to detect and classify images containing both healthy and diseased cotton plant leaves. Their approach demonstrated an average accuracy of 96% in disease identification. R. Devi Priya et al (Devi Priya et al., 2022). proposed the Augmented Faster R-CNN (AFR-CNN) algorithm by amalgamating Faster R-CNN, an efficient deep learning algorithm, with effective data augmentation techniques such as rotation, blur transformation, flipping, and GAN. The model achieved a noteworthy mAP score of 90.2%. Zhang et al (Zhang et al., 2022). introduced a real-time, high-performance detection model based on an improved YOLOX. Their model incorporated features like Efficient Channel Attention (ECA), a hard-Swish activation function, and Focal Loss into YOLOX, resulting in an mAP of 94.60% for cotton disease and pest detection, with a precision rate of 94.04%. Zhang et al (Zhang et al., 2023c). proposed an enhanced attention mechanism YOLOv7 algorithm (CBAM-YOLOv7) for the image detection of diseases and pests like cotton wilt disease. Their model achieved an impressive mean Average Precision (mAP) score of 90.2%.

The endeavors of the researchers mentioned above have undeniably advanced the field of cotton disease detection, providing valuable insights into areas such as dataset augmentation and the optimization of detection algorithms. Nonetheless, the deployment of mobile robots and various edge AI devices often necessitates a trade-off between computational power, power consumption, battery size, and the time between charges. These devices typically operate with significantly less computational power compared to the robust GPU-based systems commonly employed for training and assessing deep neural networks (Yao et al., 2022). Moreover, it has become evident that certain deep learning models with high detection accuracy tend to possess redundant model parameters. This redundancy poses challenges when it comes to deploying these models on mobile agricultural inspection robots. Existing detection models struggle to strike a balance between detection accuracy and speed, hindering their application in this context. Furthermore, it’s worth acknowledging that, in some of these studies, cotton disease detection was conducted within controlled environments, and this gap in achieving reliable detection in natural agricultural settings remains (Tang et al., 2023a). This limitation has, to a certain extent, constrained the development of agricultural inspection robots (Wang et al., 2022a; Ye et al., 2023).

Consequently, this study centered on cotton disease as the focal point of research and proposed CDDLite-YOLO detection algorithm to detect cotton disease quickly and accurately under natural field conditions. The model introduced in this paper is built upon the most recent advancements in object detection algorithms with the specific features of cotton diseases. It successfully strikes a harmonious balance between detection speed, accuracy, and model size, making it a promising candidate for deployment on an embedded GPU chip without compromising performance.

The significant contributions of this paper can be summarized as follows:

(1) We collected a dataset of cotton disease images from natural environments for training, validation, and testing of the model.
(2) To enhance detection accuracy while minimizing parameter calculations, we designed the C2f-Faster module as a replacement for the C2f module in the backbone network and introduced a novel Slim-neck structure by substituting the C2f module with the GSConv module and the VoVGSCSP module in the neck network.
(3) We introduced MPDIoU, an IoU loss measure, to address limitations for cotton disease detection that existing loss functions when predicted and ground truth bounding boxes have the same aspect ratio but varying width and height values.
(4) We designed the PCDetect detection head to reduce model parameters and computations while maintaining exceptional detection performance.
(5) Through experiments, we validated the CDDLite-YOLO model. Compared to other models, CDDLite-YOLO achieves higher mAP and detection speed, with lower FLOPs and a smaller model size.

The subsequent sections of this study are structured as follows: Section II explores critical aspects, including image acquisition, preprocessing, and model structure enhancements. Section III presents the experimental results alongside a detailed analysis, while Section IV offers a comprehensive discussion of this study. Section V encapsulates our efforts with a summary of the conclusions reached.

## 2 Materials and methods

### 2.1 Materials

#### 2.1.1 Image data acquisition

The image dataset was collected from two specific locations: the cotton fields at the Langfang Research Base of the Chinese Academy of Agricultural Sciences, Hebei Province, China (N: 39°27′55.59″, E: 116°45′28.54″), and the Potianyang Base in Yazhou District, Sanya City, Hainan Province, China (N: 18°23′49.71″, E: 109°10′39.84″). This data collection took place from September 2020 to December 2022.The focus of our image collection comprised three primary types of cotton diseases: verticillium wilt, fusarium wilt, and anthracnose. To ensure the quality and accuracy of the dataset, all images underwent a meticulous identification and confirmation process carried out by two expert cotton pathologists.

Images were captured during different weather conditions, including clear and overcast skies, at various times of the day, covering the morning, noon, and evening. Image capture was carried out using a Canon EOS 850D digital camera (Canon Inc., Tokyo, Japan) and a Huawei P40 Pro smartphone (Huawei Technologies Co., Ltd., Shenzhen, China). The images were captured from a distance of 20–50 cm from the cotton leaves, using automatic exposure mode. They have a resolution of 4608 × 3456 pixels and were saved in JPG format.

To ensure the diversity and richness of our image dataset, a randomized approach was employed during the collection process. This involved capturing images from various angles, under different lighting conditions, and against diverse backgrounds. To accurately reflect natural field conditions, images were taken during different weather conditions, including sunny, cloudy, and overcast weather, across different times of the day, encompassing various growth stages of the cotton crop. The images also include the presence of soil, as well as potential field clutter such as weeds, plastic film, and dried leaves.

#### 2.1.2 Images processing and dataset production

To enhance data collection efficiency, we concurrently captured images and recorded videos. Later, we employed video frame extraction to augment the image count. The recorded videos ranged from 15 to 30 seconds, and frames were extracted at a rate of 15 frames per second, resulting in a range of 225 to 450 frames, and the image resolution is 4608 × 3456, which is saved in JPG format. These frames were then carefully curated for selection. In order to prevent redundancy within the dataset, we adhered to three guiding principles for image selection: (1) ensuring each diseased leaf was represented only once, (2) avoiding multiple images from the one or neighboring cotton plants, and (3) prioritizing images with different angles, various lighting conditions, and diverse backgrounds. Consequently, we curated a dataset for cotton disease detection under natural conditions, comprising 591 images of cotton with verticillium wilt, 435 images of cotton with fusarium wilt, and 504 images of cotton with anthracnose, totaling 1,530 images. For specific details regarding the types of cotton diseases, the number of images in each category, and key disease features within the dataset, please refer to **Table 1**.

**Table 1:** The types, figures, image samples, and key features of each cotton disease in the dataset.

Type of Disease	Figures	Image	Key Features
Verticillium wilt	591	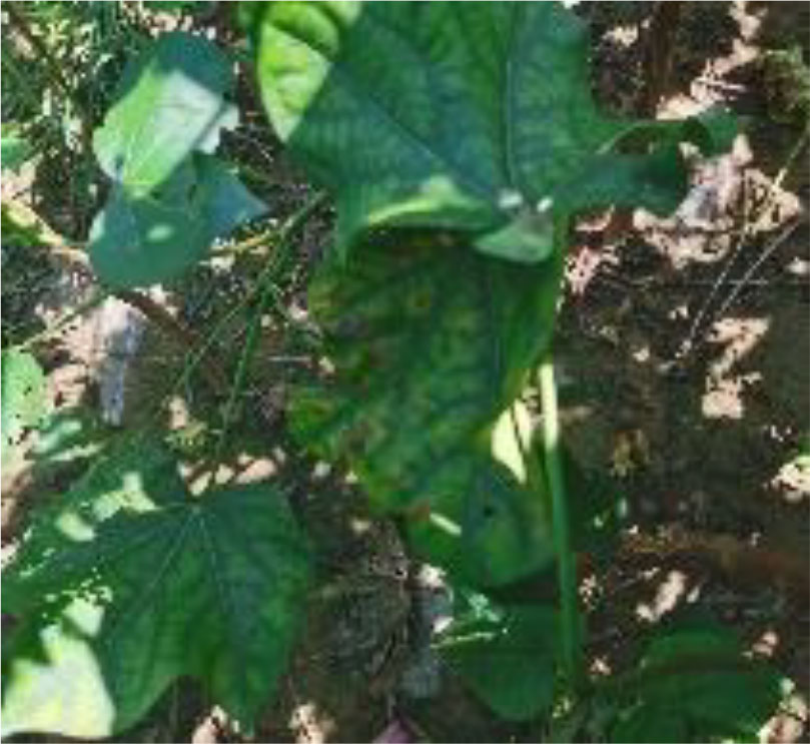	Pale yellow patches develop between leaf margins and veins, gradually expanding and causing the loss of green color in the leaves.
Fusarium wilt	435	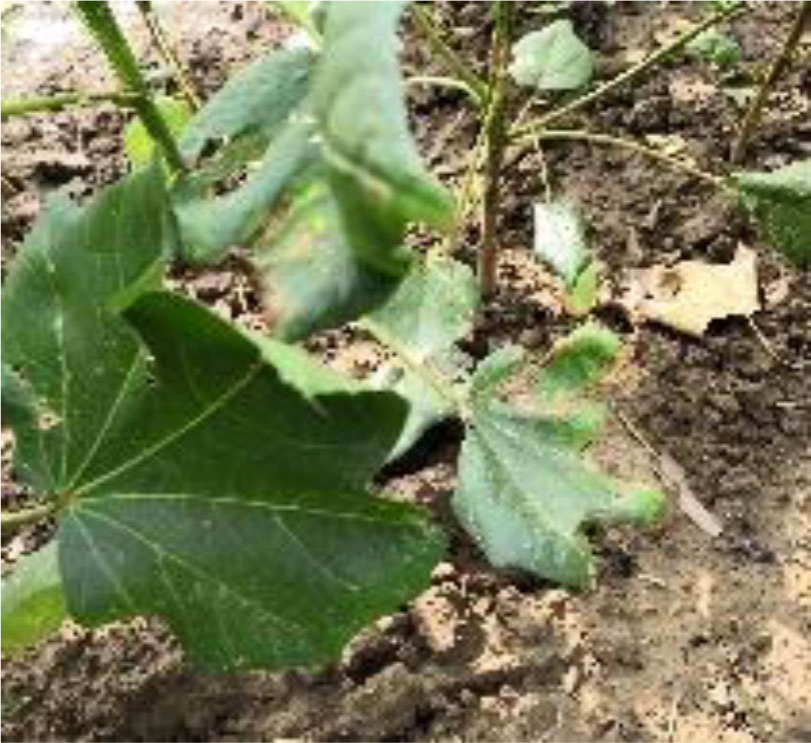	Lower leaves exhibit yellowing and wilting. The stem displays brown discoloration and often splits open, revealing red-brown vascular tissue.
Anthracnose	504	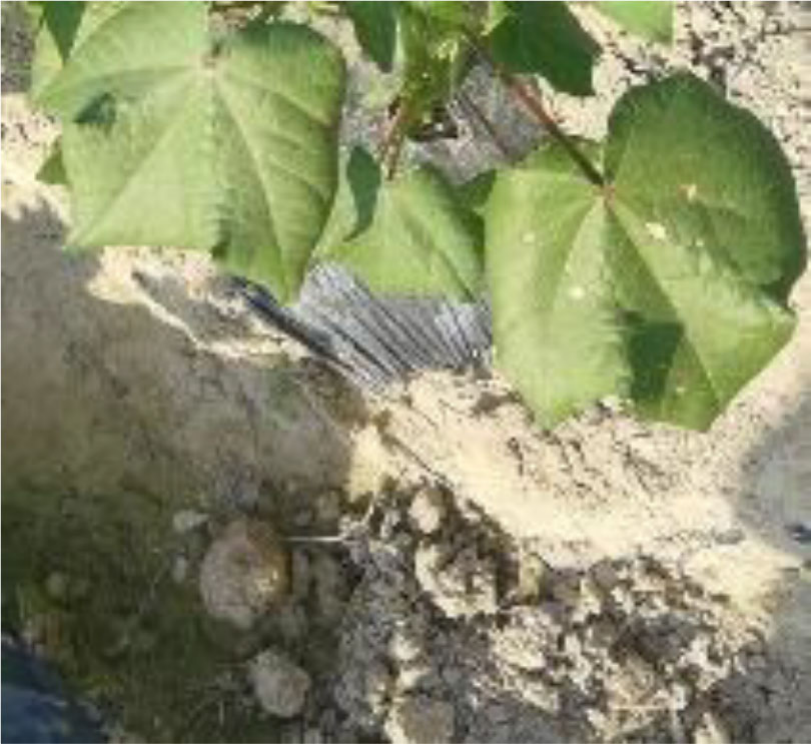	Small, circular lesions appear on leaves, stems, and bolls. These lesions start as water-soaked areas and become sunken with dark centers over time.
Total	1530		

We employed the Make Sense tool (https://makesense.ai) for labeling the types of diseased leaves and their respective positions in the images. The labeling area was defined as the smallest rectangle encompassing the diseased leaf, minimizing background inclusion. The dataset was partitioned into three subsets in an 8:1:1 ratio, with 1224 images allocated to the training set, and 153 images each for both the validation and test sets. Additionally, mosaic augmentation was incorporated into the training process. Mosaic augmentation randomly selects four images, extracting segments of content and their corresponding detection box information. These segments are then fused into a single image for network input. This method substantially enhances training data diversity, mitigating the risk of overfitting by introducing greater variability into the learning process.

### 2.2 Methods

#### 2.2.1 Overall model

**Figure 1:**
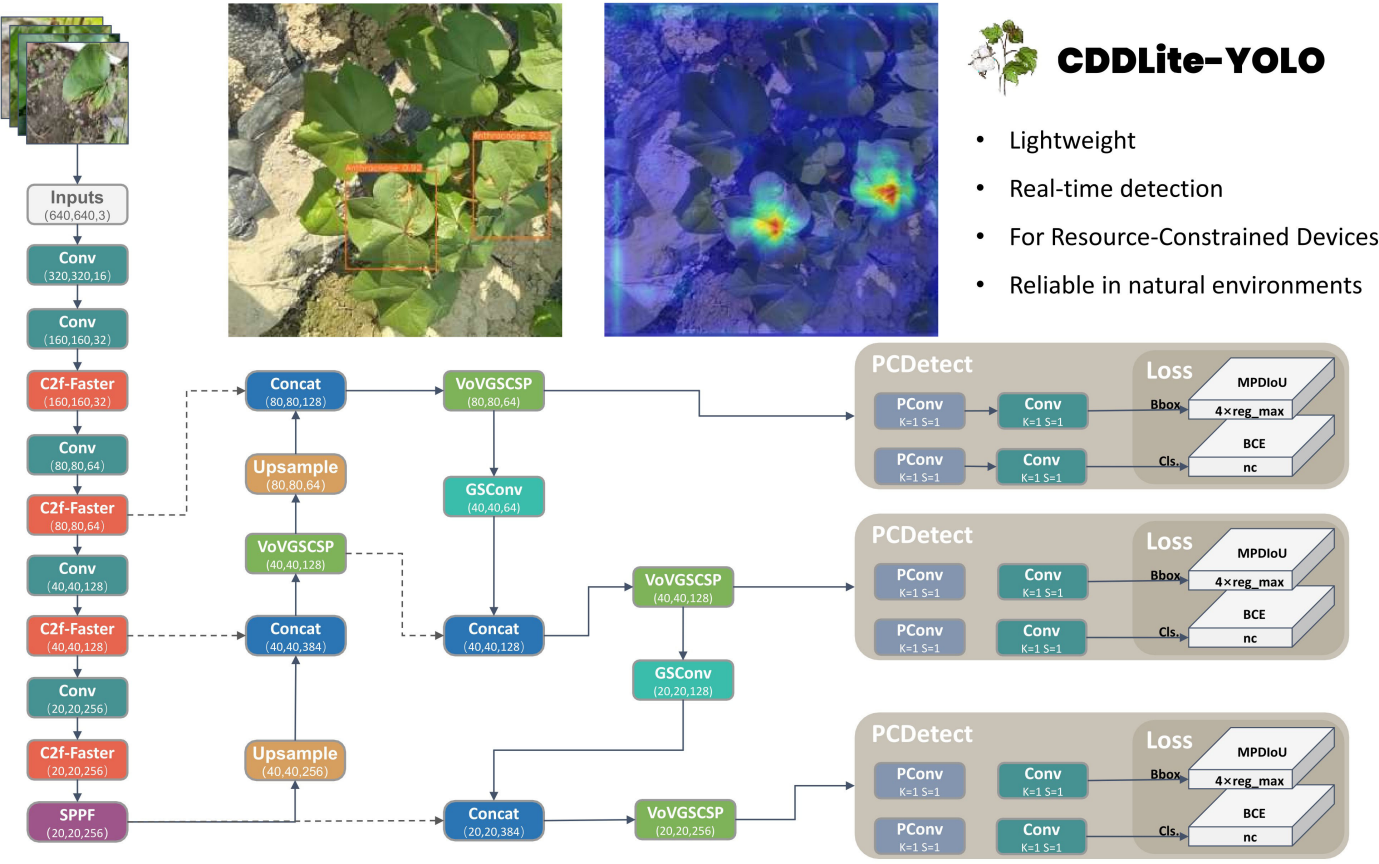
Overall model architecture diagram.

Object detection algorithms can be categorized into one-stage and two-stage algorithms. The two-stage algorithm relies on region proposals, represented by Faster R-CNN, which is known for its slower processing speed, which makes it unsuitable for real-time detection and deployed on an embedded GPU chip. On the other hand, the one-stage model is based on regression, which includes the YOLO series. offers a significant advantage in speed compared to the two-stage model, making it better suited for real-time detection requirements. Hence, this study opts for the YOLO model as the baseline model. This model is an enhancement of the YOLOv8 model specifically tailored for the task of detecting cotton diseases in natural environments and designed for deployment on agricultural inspection robots and other devices with limited memory and computational resources. The architecture of CDDLite-YOLO is visualized in **Figure 1**.

The model comprises four key components: Input, Backbone, Neck, and Head. The enhancements are summarized as follows:

(1) We designed the Faster Block structure using partial convolution to replace the Bottleneck structure in the C2f module within the backbone network, resulting in the upgraded C2f module termed C2f-Faster.
(2) In the neck network, we introduce an innovative Slim-neck structure by replacing the C2f module with the GSConv module. Additionally, the C2f modules are enhanced by integrating the VoVGSCSP module. This module is an iterative fusion of the GS bottleneck, built upon GSConv.
(3) We introduced MPDIoU, an IoU loss function based on minimum points distance, to address limitations in existing loss functions in YOLOv8, particularly when dealing with predicted and ground truth bounding boxes of the same aspect ratio but varying width and height values.
(4) We designed the PCDetect detection head, incorporating the PCD module into the detection head and replacing specific CBS modules with PCDetect.

By integrating these advancements, CDDLite-YOLO effectively balances detection speed, accuracy, and model size. It significantly reduces the model’s size, accelerates detection speed, and achieves higher detection accuracy, providing a harmonious synergy of performance improvements.

#### 2.2.2 YOLOv8

YOLOv8, the latest addition to the YOLO series, was introduced by Ultralytics in January 2023. It maintains the established YOLO series structure while undergoing significant optimization, resulting in notable improvements in both speed and accuracy (Kang and Kim, 2023).

YOLOv8 consists of three core components: Backbone, Neck, and Head. The Backbone in YOLOv8 closely mirrors YOLOv5’s architecture, with notable refinements to the CSPLayer, now referred to as the C2f module. This C2f module seamlessly integrates high-level features with contextual information, resulting in improved detection accuracy. The Neck of YOLOv8 combines an FPN (Feature Pyramid Network) and PAN (Path Aggregation Network) to facilitate feature fusion among the three effective feature layers obtained in the Backbone. In the Head of YOLOv8, a shift occurs from an anchor-based to an anchor-free approach (Terven and Cordova-Esparza, 2023). This transition abandons IOU matching and single-scale assignment, opting instead for a task-aligned assigner to match positive and negative samples.

YOLOv8n, the smallest model in the YOLOv8 series, is distinguished by its compact model parameters and minimal hardware requirements. When trained on the cotton diseases dataset, YOLOv8n surpasses the performance of YOLOv8s, YOLOv8m, YOLOv8l, and YOLOv8x, yielding notably superior results. Although its mAP value is slightly lower compared to the other four models, YOLOv8n shines with significantly reduced computational costs and fewer parameters. This renders it an optimal choice for deployment on resource-constrained devices.

In this article, we present the CDDLite-YOLO model, built upon YOLOv8n. Our objective is to cater to real-time and resource-constrained device development requirements while upholding detection accuracy in natural field environments.

#### 2.2.3 C2f-faster

In object detection models, the main objective is to extract spatial information from images, which demands a substantial number of convolutional operations. In contrast to YOLOv5’s C3 module, YOLOv8’s new C2f module incorporates additional Bottleneck structures and cross-layer connections, enhancing gradient flow. However, this also brings about excessive convolution operations and heightened computational load, presenting deployment challenges on resource-limited embedded devices.

To meet the requirements of embedded devices for cotton disease detection, reduce computational complexity, and minimize parameter size, thus achieving a lightweight network model, enhancing the convolution operator within the C2f module stands out as a highly effective and worthwhile approach.

**Figure 2:**
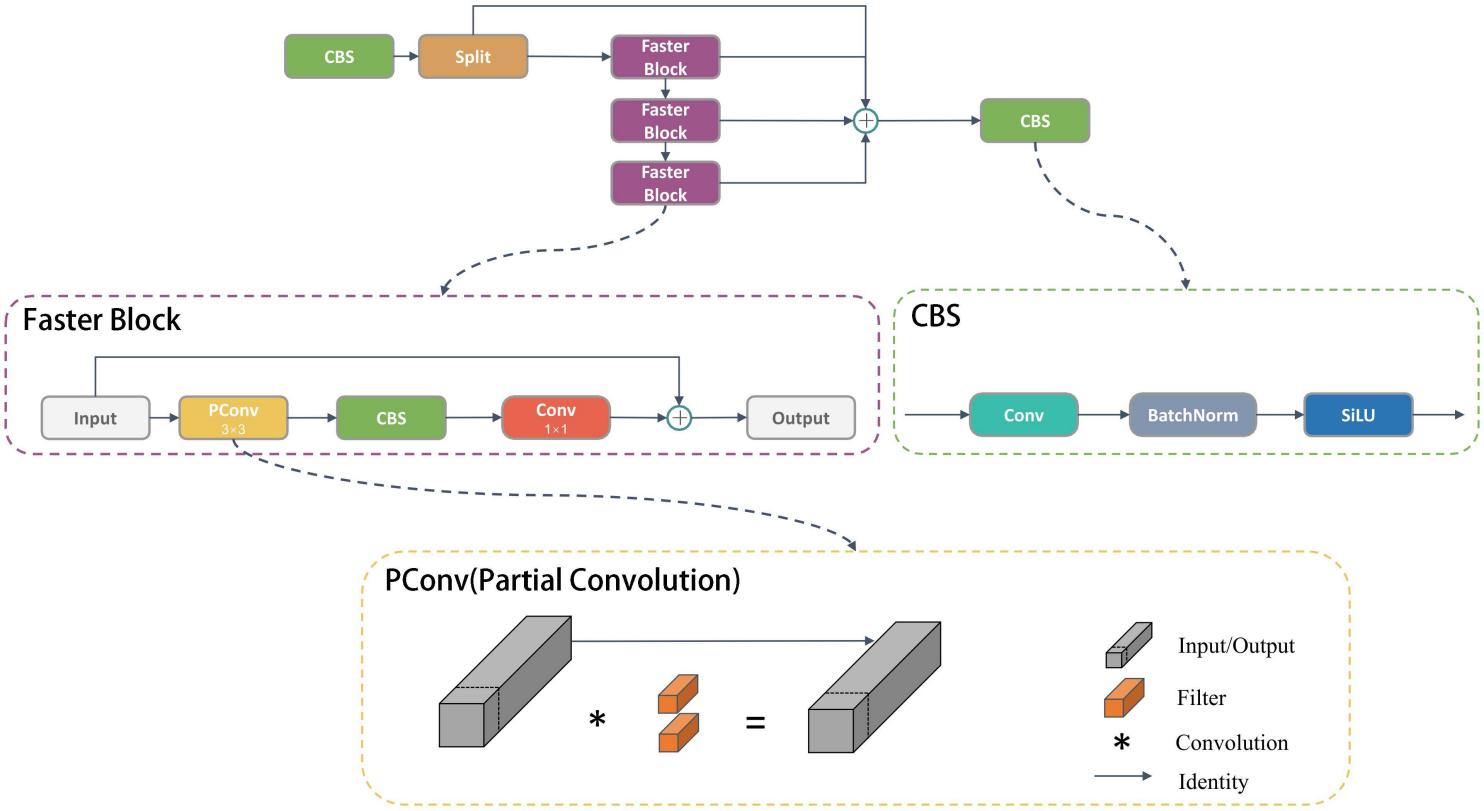
Structural diagram of C2f-Faster.

The feature maps exhibit significant similarities across various channels. FasterNet (Chen et al., 2023) introduced the concept of partial convolution, where it applies a regular Conv operation to only a subset of the input channels for spatial feature extraction, leaving the rest unchanged. This approach reduces computational redundancy and memory usage simultaneously, resulting in efficient performance on a wide range of devices. The C2f-faster module is inspired by the lightweight design principles of FasterNet. It utilizes the Faster Block to replace the Bottleneck within the C2f module, as illustrated in the **Figure 2**.

The Faster Block encompasses three types of blocks: PConv, CBS, and 1×1 Conv. PConv stands for Partial Convolution, and utilizes only 1/4 of the input channels for convolution, leaving the remaining 3/4 channels untouched. The outputs of the convolved 1/4 channels are then merged with the untouched 3/4 channels. For contiguous or regular memory access, the first or last consecutive cp channels as the representatives of the whole feature maps for computation. Without loss of generality, we assume the input and output feature maps have the same number of channels, which aims to reduce redundant calculations while preserving the original channel information. Despite 3/4 of the channels not being involved in convolution, they are not discarded. Instead, valuable information can be extracted from these channels in subsequent 1×1 convolutions. This approach enhances the efficiency of spatial feature extraction by reducing redundant computation and memory access concurrently. Additionally, CBS is composed of Conv, batch normalization, and a SILU activation function. To ensure that the processed feature maps maintain their original dimensions and size, the 1×1 Conv layer is utilized to restore the output of the preceding layer.

#### 2.2.4 Slim-neck

The standard convolution (SC) module in YOLOv8 utilizes different convolutional kernels across multiple channels simultaneously, resulting in a higher parameter count and increased computational requirements (FLOP). While lightweight networks like MobileNet (Howard et al., 2017) and ShuffleNet (Zhang et al., 2018) effectively address this issue using Depth-wise Separable Convolutions (DSC), they suffer from reduced feature extraction and fusion capabilities, hindering model detection performance. Such limitations make them unsuitable for real-time cotton disease detection.

To address these challenges, the CDDLite-YOLO model introduces the GSConv module (Li et al., 2022), a lightweight convolution, into the neck section, resulting in a novel Slim- neck structure. The GSConv module utilizes the shuffle operation to seamlessly integrate information from SC into DSC-generated data. In contrast to DSC, GSConv excels at preserving hidden connections while still keeping complexity low, achieving a balanced trade-off between model accuracy and speed.

**Figure 3:**
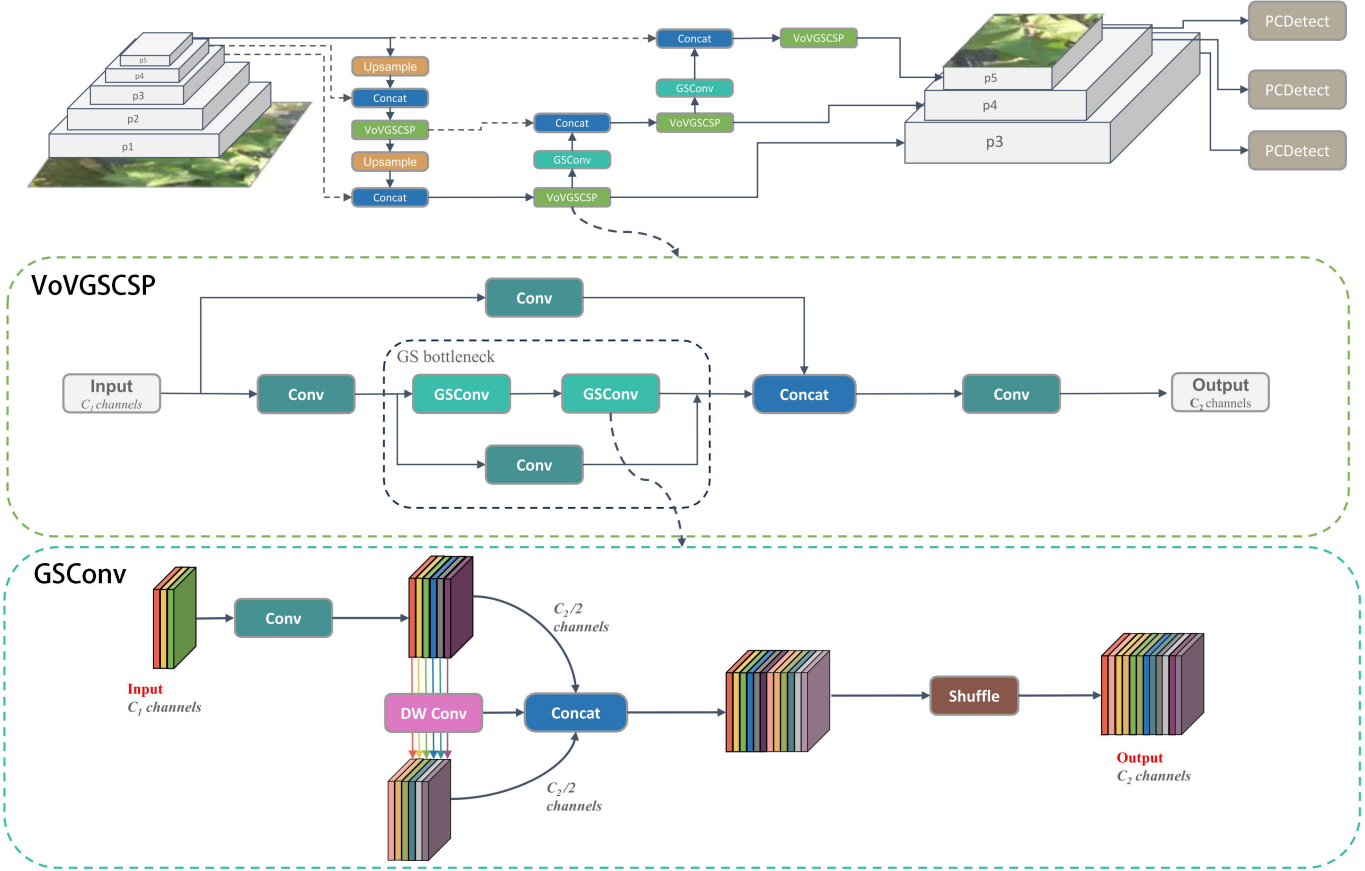
Structural diagram of neck.

The GSConv module is primarily constituted by Conv, DWConv, Concat, and Shuffle operations, visually represented in the **Figure 3**. The construction unfolds as follows:

(1) The input feature map consists of C1 channels.
(2) Half of the channels undergo Standard Convolution (SC), and the remaining half undergo Depthwise Separable Convolution (DSC).
(3) Concatenate the resulting two output feature maps along the channel dimension.
(4) Subject the concatenated feature map to a shuffle operation, resulting in the final output.
(5) The final output feature map now contains C2 channels in total.

VoVGSCSP (Xu et al., 2023) represents an iterative integration that builds upon the GS bottleneck using the foundation of GSConv, as depicted in **Figure 3**. This process involves segmenting the input feature map’s channel count into two portions. The initial segment undergoes Convolution (Conv) for processing, followed by consecutive GS bottleneck modules for feature extraction. Simultaneously, the remaining segment serves as residuals and undergoes a single Convolution operation. The resulting two output feature maps are then concatenated and subjected to an additional Convolution, resulting in the final output. The ultimate output feature map contains a total of C2 channels. This module effectively strikes a balance between model accuracy and speed, concurrently reducing computational load and complexity while preserving commendable accuracy.

We envisioned integrating GSConv and VoVGSCSP into the neck network to create a lightweight model without compromising detection performance, as illustrated in the **Figure 3**. This enhancement led to a reduction in model parameter calculations, fostering high detection accuracy and a notable improvement in the balance between the model’s accuracy and speed.

#### 2.2.5 MPDIoU

Computing the loss involves comparing the network’s predicted results with the groundtruth (Tian et al., 2022). Our model’s loss function aligns with YOLOv8, encompassing regression and classification components. YOLOv8 utilizes DFL and CIoU for bounding box regression (Xiao et al., 2023).

The training dataset comprises precisely ground truth bounding boxes that accurately delineate diseased areas. In cotton disease detection, the diverse range of diseases, variations across growth stages, and the influence of factors such as camera angles, lighting conditions, and obstructions can introduce discrepancies in disease localization.However, the aspect ratio definition in CIoU is relative rather than absolute. In instances where predicted and ground truth bounding boxes share the same aspect ratio but differ in width and height, the model may generate boxes with slight deviations (Zhang et al., 2023d). CIoU’s sensitivity to such nuances poses challenges for precise learning and prediction, impacting convergence speed and accuracy. To mitigate this, we introduced a novel bounding box similarity comparison metric, MPDIoU (Siliang and Yong, 2023), based on the minimum point distance.

MPDIoU incorporates three key factors: overlapping or non-overlapping area, central points distance, and width and height deviation. It streamlines calculations by minimizing the distance between top-left and bottom-right points in predicted and ground truth bounding boxes. This adaptable metric accommodates overlapping or non-overlapping bounding box regression. Equation 1 shows the computation method for MPDIoU.


(1) $$\begin{array}{c} d_{1}^{2}={\left(x_{1}^{B}-x_{1}^{A}\right)}^{2}+{\left(y_{1}^{B}-y_{1}^{A}\right)}^{2}\\ d_{2}^{2}={\left(x_{2}^{B}-x_{2}^{A}\right)}^{2}+{\left(y_{2}^{B}-y_{2}^{A}\right)}^{2}\\ MPDIoU=\frac{A\cap B}{A\cup B}-\frac{d_{1}^{2}}{w^{2}+h^{2}}-\frac{d_{2}^{2}}{w^{2}+h^{2}} \end{array}$$

In the formulation, $d_{1}$ and $d_{2}$ represent the intersection and minimum point distance. Shapes A and B are two arbitrary convex entities, with w and h signifying the width and height of the input image. The coordinates ( $x_{1}^{A}$ , $y_{1}^{A}$ ) and ( $x_{2}^{A}$ , $y_{2}^{A}$ ) denote the top-left and bottom-right points of shape A, respectively, and ( $x_{1}^{B}$ , $y_{1}^{B}$ ) and ( $x_{2}^{B}$ , $y_{2}^{B}$ ) represent the top-left and bottom-right points of shape B.

Benefiting from the implementation of MPDIoU to replace CIoU in YOLOv8, our model has demonstrated competitive results. The subsequent section detailing illustrates that our proposed MPDIoU surpasses the original CIoU and other loss functions.

#### 2.2.6 PCDetect

YOLOv8 introduces the decoupled head mechanism, separating convolutional layers from fully connected layers. This technique leverages neck network output features to predict category and location via distinct branches. While enhancing model convergence and accuracy, the decoupling head introduces additional parameters and computational costs.

To boost computational efficiency, we propose the PCD module, building on PConv from Section 2.2.3. The PCD module features a 3 × 3 PConv layer for extraction, augmented by a CBS module using a 1×1 convolutional kernel for channel adjustment. This enhancement improves feature fusion and cross-channel perception without a substantial parameter increase, enhancing model expressiveness.

The PCD module replaces some CBS modules in the detection head, forming PCDetect. Input and output feature maps are H × W × C. Equation 2 shows the FLOPs ratio of PCD to traditional convolution is only 1/5–1/6 when k = 3, r = 4 (Jiang et al., 2023).


(2) $$s=\frac{FLOPs_{PCD}}{FLOPs_{Conv}}=\frac{k\times k\times C/r\times W\times H\times C/r+C\times W\times H\times C}{k\times k\times C\times H\times W\times C}=\frac{1}{r^{2}}+\frac{1}{k^{2}}\;$$

Substituting PCDetect for the Detection module in YOLOv8 significantly reduces model parameters while maintaining similar detection accuracy. This effectively resolves conflicts between accuracy and detection speed.

## 3 Experiments and analysis of results

### 3.1 Experiment settings

#### 3.1.1 Experimental parameter settings

The experimental setup utilized a Dell tower workstation (Dell, Inc., Round Rock, Texas, USA) running Windows 11. It was equipped with a 12th Gen Intel(R) Core(TM) i5–12500 processor operating at 3.00 GHz, 32GB of RAM, a 1TB solid-state drive, and an NVIDIA GeForce RTX 3080 graphics card with 10GB of video memory for GPU-accelerated computing. The software environment included Python 3.8.17, PyTorch 1.13.0, Torchvision 0.14.0, and CUDA 11.7.

The experiment comprised 300 iterations with a batch size of 4. The optimization algorithm used was Adam, with an initial learning rate of 1e-3, a maximum learning rate of 1e-5, a momentum of 0.937, a weight decay of 5e-4, and an input image resolution of 640×640. These training parameters and dataset were consistent across all models during the training process.

#### 3.1.2 Evaluation indicators

To assess the model’s performance, various evaluation metrics were used, including Precision, Recall, mAP@0.5, mAP@0.5:0.95, Speed (measured in frames per second or FPS), the number of parameters (Params), and computation costs (FLOPS).

Precision measures the ratio of correctly classified positive samples to all samples predicted as positive, calculated using the formula in Equation 3:


(3) $$Precision=\frac{TP}{TP+FP}$$

Where TP is the true positive samples, and FP is the false positive samples.

Recall quantifies the proportion of actual positive samples correctly identified by the model, calculated using Equation 4:


(4) $$Recall=\frac{TP}{TP+FN}$$

mAP, which stands for mean Average Precision, is determined through the precision-recall (PR) curve and is calculated using Equation 5:


(5) $$mAP=\frac{\sum_{i=1}^{N}APi}{N}$$

Where mAP@0.5 is the average AP with an IoU of 0.5, and mAP@0.5:0.95 is the average AP with IoU values ranging from 0.5 to 0.95 in steps of 0.05.

The number of parameters (Params) reflects the model’s complexity and its capacity to learn and represent features. It’s calculated using Equation 6:


(6) $$\text{Param }=\sum \left(K\times K\times C_{\text{in }}\times C_{\text{out }}\right)$$

Where K represents the convolution kernel size, C*_in_* is the input size, and C*_out_* is the output size.

Speed is measured in frames per second (FPS), calculated using Equation 7:


(7) speed=frames/time

FLOPS (Floating-Point Operations Per Second) represents the model’s computation costs, and its calculation is detailed in Equation 8:


(8) $$FLOPs=\sum \left(K\times K\times C_{\text{in }}\times C_{\text{out }}\times H\times W\right)$$

Where H × W is the size of the outputted feature map.

### 3.2 Analysis of results

#### 3.2.1 Ablation experiments

For a more in-depth evaluation of the effectiveness of the enhancement technique in the CDDLite-YOLO model, we performed a series of ablation experiments. We used YOLOv8 as the baseline model for comparison, and the results can be found in **Table 2**.

(1) Effects of C2f-Faster: A comparative analysis between YOLOv8 and experiments involving the gradual addition of the C2f-Faster module highlights its effectiveness. The incorporation of C2f-Faster significantly reduces computational costs, with a 13.41% reduction in FLOPS and a 13.33% decrease in Params. Simultaneously, it modestly enhances feature extraction capabilities, leading to a 1.3% increase in mAP@0.5. This demonstrates that C2f-Faster not only significantly reduces parameters but also reduces computational costs without compromising detection accuracy.
(2) Effects of Slim-neck: A comparison between YOLOv8 and experiments involving the gradual integration of the Slim-neck module reveals that the inclusion of the Slim-neck contributes to a reduction in computational costs. It leads to a notable 10.98% reduction in FLOPS and a 10.00% decrease in Params. Simultaneously, it provides a modest enhancement in feature extraction capabilities, resulting in a 1.4% increase in mAP@0.5. When both C2f-Faster and Slim-neck are added, computational costs experience a significant decrease, with FLOPS and Params decreasing by 24.39% and 20.00%, while mAP@0.5 remains stable. This achieves model lightweight without compromising mAP@0.5. This outcome can be primarily attributed to the incorporation of the GSConv and VoVGSCSP module, which utilizes depthwise separable convolution to significantly reduce the number of computed parameters. Additionally, it reshuffles the connections between channels to ensure information multiplexing, thereby maintaining detection accuracy. The deliberate decision to integrate the GSConv module into the neck was made with careful consideration. However, it was intentionally omitted from the backbone to prevent an excessive presence of GSConv modules. This choice aimed to avoid over-complicating the network architecture, which could hinder the flow of spatial information and substantially increase inference times.
(3) Effects of MPDIoU: A comparative analysis between YOLOv8 and experiments gradually introducing the MPDIoU module highlights the efficacy of its integration. The addition of MPDIoU notably enhances model accuracy, achieving a mAP@0.5 of up to 90.7% and showing improvements of 2.10%, with no additional parameters and speed costs. It also achieves high accuracy when integrated with other improvements. This substantiates that MPDIoU indeed contributes to improved model performance by calculating the IoU based on minimizing the point distance between the predicted bounding box and the ground truth bounding box.
(4) Effects of PCDetect: A comparative analysis between YOLOv8 and experiments involving the gradual addition of the PCDetect module highlights its effectiveness. The incorporation of PCDetect contributes to a reduction in computational costs, with FLOPS and Params experiencing reductions of 31.71% and 20.00%, respectively. It maintains accuracy while achieving these reductions when integrated with other improvements.
(5) Effects of integrating together: CDDLite-YOLO seamlessly combines the strengths of C2f-Faster, Slim-neck, MPDIoU, and PCDetect. The result is a model with a 56.10% reduction in parameters, a 40.00% decrease in computational demand, and a noteworthy 2.00% improvement in mAP@0.5 compared to YOLOv8.

**Table 2:** Comparisons of ablation experiments.

BaseLine	C2f-Faster	Slim- neck	MPDIoU	PCDetect	mAP@0.5	FLOPS/G	Params/M
✓					88.6%	8.2	3.0
✓	✓				89.9%	7.1	2.6
✓	✓	✓			89.3%	6.2	2.4
✓	✓	✓	✓		90.2%	6.2	2.4
✓		✓			90.0%	7.3	2.7
✓		✓	✓		90.1%	7.3	2.7
✓		✓	✓	✓	89.6%	4.7	2.2
✓			✓		90.7%	8.2	3.0
✓			✓	✓	89.4%	5.6	2.4
✓				✓	89.0%	5.6	2.4
**✓**	**✓**	**✓**	**✓**	**✓**	**90.6%**	**3.6**	**1.8**

The bold values in **Table 2** represent the model proposed in this paper.

The CDDLite-YOLO model significantly reduces both model size and computational costs while maintaining a comparable detection accuracy. This emphasizes a harmonious balance between enhancing accuracy and streamlining model efficiency, underscoring the significance of our proposed improvements.

#### 3.2.2 Performance comparison with the state-of-the-art detection models

To evaluate the model’s effectiveness, we conducted comparative experiments, comparing our proposed model against well-known lightweight models such as YOLOv5n, YOLOv6n, YOLOv7-tiny, and YOLOv8n. All experiments utilized the same cotton diseases dataset, which consists of 1224 training images, 153 validation images, and 153 test images. We maintained identical experimental conditions throughout to ensure a fair comparison.

**Figure 4:**
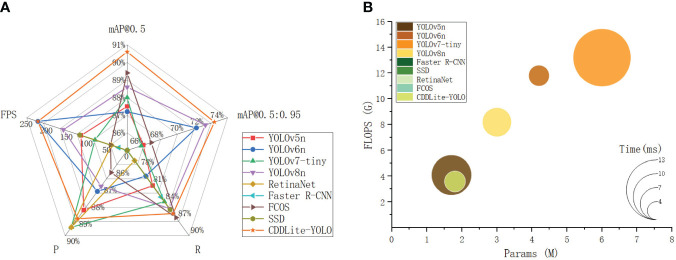
Comparison of detection results between different models. **(A)** Detection performance. **(B)** Computational complexity, parameter, and detection time.

The comparison results are shown in **Figure 4** and **Table 3**.

**Table 3:** Comparison of detection performance of different models.

Models	Precision	Recall	mAP@0.5	mAP@0.5:0.95	FLOPS/G	Params/M	Speed
YOLOv5n	88.5%	81.2%	87.5%	66.6%	4.1	1.7	114.9
YOLOv6n	87.4%	79.5%	87.2%	71.9%	11.8	4.2	220.3
YOLOv7-tiny	89.5%	84.0%	88.0%	66.4%	13.2	6.0	80.0
YOLOv8n	87.1%	85.2%	88.6%	72.8%	8.2	3.0	158.7
Faster R-CNN	43.9%	83.2%	74.3%	46.3%	370.2	137.1	21.0
SSD	74.4%	85.4%	82.1%	58.4%	62.8	26.3	117.6
RetinaNet	89.5%	76.8%	84.4%	60.8%	170.1	38.0	39.3
FCOS	86.3%	86.8%	89.4%	67.4%	161.9	32.2	41.5
CDDLite-YOLO	89.0%	86.1%	90.6%	73.7%	3.6	1.8	222.2

CDDLite-YOLO outperforms other mainstream lightweight models in terms of detection accuracy. In this paper, CDDLite-YOLO achieves mAP@0.5 and mAP@0.5:0.95 scores of 90.6% and 73.7%, surpassing the performance of YOLOv5n, YOLOv6n, YOLOv7-tiny, Faster R-CNN, SSD, RetinaNet, FCOS and YOLOv8n. Several factors contribute to this superior performance. Firstly, the C2f-Faster module utilizes only 1/4 of the input channels for convolution and processing 3/4 of the channels extracted from these channels in subsequent 1×1 convolutions. This approach enhances spatial feature extraction by reducing redundant computation and memory access simultaneously. Secondly, Slim-neck utilizes the shuffle operation to seamlessly integrate information from SC into DSC-generated data while preserving hidden connections. This approach effectively achieves a balanced trade-off between model accuracy and speed, keeping complexity low. Additionally, the PCDetect module employs a 1×1 convolutional kernel for channel adjustment, enhancing feature fusion and cross-channel perception without substantially increasing parameters. The integration of the C2f-Faster module, Slim-neck, and PCDetect module significantly reduces operational parameters while maintaining inference speed, without compromising detection accuracy. Furthermore, the inclusion of MPDIoU is pivotal in enhancing model accuracy. It addresses limitations in existing loss functions by considering the minimum point distance between predicted and ground truth bounding boxes, particularly when they share the same aspect ratio but possess varying width and height values. These factors collectively enhance the effectiveness of the CDDLite-YOLO model in detecting cotton diseases.

The CDDLite-YOLO model excels in reducing parameter count and computational complexity. Compared to YOLOv5n, YOLOv6n, YOLOv7-tiny, Faster R-CNN, SSD, RetinaNet, FCOS and YOLOv8n, our proposed CDDLite-YOLO model offers lower FLOPS and Params, specifically 3.6G and 1.8M. This reduction can be mainly attributed to the incorporation of the C2f-Faster module, Slim-neck, and PCDetect module.

Upon analyzing the results, we observe that the Params of the YOLOv5n model are slightly lower than those of our proposed model, albeit by only 0.1. However, what sets CDDLite-YOLO apart is its superior performance in terms of Precision, Recall, mAP@0.5, mAP@0.5:0.95, and speed. The CDDLite-YOLO model outperforms YOLOv5n with a 0.5% increase in Precision, 4.9% in Recall, 3.1% in mAP@0.5, 7.1% in mAP@0.5:0.95, and a remarkable 107.28 FPS boost in speed.

**Figure 5:**
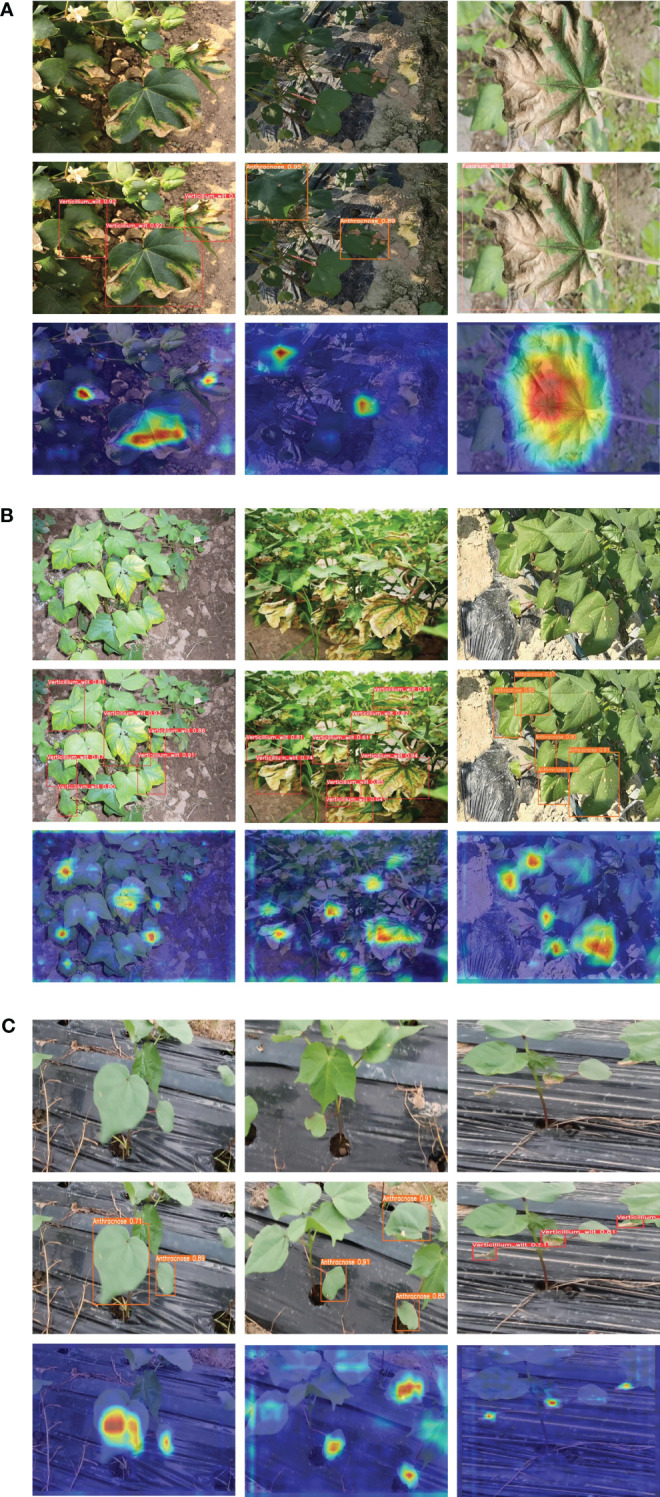
Prediction results of the proposed method. **(A)** Under complex backgrounds such as plastic film, water pipes, and soil in the field. **(B)** Under dense disease conditions. **(C)** Under the conditions of image blurriness generated during the agricultural inspection robot movement and collection process.

The results unequivocally establish the superiority of our proposed model over the current mainstream lightweight algorithms in three key aspects: model size, detection accuracy, and detection speed. To further substantiate the performance of the CDDLite-YOLO model, we randomly selected detection results from a variety of environmental conditions among all testing samples, as displayed in **Figure 5**.

#### 3.2.3 Performance comparison of loss function

We experimented with various IoU loss functions to determine their impact on performance. The tested loss functions include CIoU loss, GIoU loss (Rezatofighi et al., 2019), SIoU loss (Gevorgyan, 2022), WIoU loss (Cho, 2021), and MPDIoU loss, while the remaining aspects of the YOLOv8 model were kept constant. The comparative results are presented in the **Table 4**.

**Table 4:** Comparison of different loss functions onYOLOv8.

Loss Functions	CIoU (Origin YOLOv8)	GIoU	SIoU	WIoU	MPDIoU
mAP@0.5	88.6%	89.3%	90.1%	90.0%	90.7%

Notably, when using MPDIoU as the loss function for YOLOv8, the highest mAP is achieved. This can be attributed to its adaptability to diseases of various shapes and sizes in field environments, distinguishing it as the most suitable choice for our model in comparison with the other tested loss functions, particularly when compared to the original IoU loss.

#### 3.2.4 Performance comparison of detection head optimization

To evaluate the impact of the PCDetect detection head on cotton disease detection, we conducted experiments to determine the most effective detection head. We tested several detection heads, including Origin YOLOv8 (featuring two 3x3 Conv layers), a detection head with one 1x1 ScConv (Li et al., 2023a) + one 1x1 Conv, a detection head with two 3x3 RepConv (Soudy et al., 2022), and PCDetect (comprising 1x1 PConv + one 1x1 Conv). The Comparison of different detection heads on YOLOv8 is shown in **Table 5**.

**Table 5:** Comparison of different detection heads onYOLOv8.

Detection head	Origin YOLOv8	one 1x1 ScConv + one 1x1 Conv	two 3x3 RepConv	PCDetect
mAP@0.5	88.6%	88.7%	89.6%	89.0%
FLOPS/G	8.2	5.7	8.1	5.6
Params/M	3.0	2.5	3.8	2.4

Comparing PCDetect with Origin YOLOv8 and the detection head with one 1x1 ScConv + one 1x1 Conv, we observed that the mAP@0.5 of the PCDetect detection head remained stable. However, the number of parameters decreased by 20% and 4%, while computational complexity increased by 31.71% and 1.75%. It’s worth noting that although the mAP@0.5 of the detection head with two 3x3 RepConv was 0.6 higher than that of PCDetect, the computational costs and parameter count increased by 44.64% and 58.33% compared to PCDetect, even surpassing those of the Origin YOLOv8 model.

Our experimental results unequivocally confirm that using the PCDetect detection head outperforms other options, maintaining detection accuracy while requiring fewer parameters and lower computational complexity.

## 4 Discussion

### 4.1 The importance of model lightweight

In recent years, advances in deep learning and convolutional networks have significantly enhanced object detection capabilities. Embedded computing devices have emerged as the preferred computational core for cost-effective and portable agricultural equipment. However, a graphics card’s performance depends on its single-precision floating-point capabilities, CUDA core count, and overall computing power, creating a noticeable power gap between embedded devices and professional computing cards (Cui et al., 2023). Consider the NVIDIA H100, a pinnacle in professional computing, with an impressive 1200.00 TFlops in single-precision floating-point performance and a substantial 18432 CUDA cores. Meanwhile, the NVIDIA A100, another powerhouse in professional computing, maintains a balanced profile with 312.00 TFlops and 6912 CUDA cores. On the other hand, the NVIDIA GeForce RTX 4090, a robust GPU not specifically tailored for professional computing, emphasizes a different performance profile with 82.58 TFlops and 16384 CUDA cores. In contrast, embedded devices like the NVIDIA Jetson AGX Orin and Jetson TX2, efficient in their own right, demonstrate more modest capabilities with 5.30 TFlops/2560 CUDA cores and 1.36 TFlops/256 CUDA cores, respectively.

Deep learning models demand a considerable number of multiplicative operations for accurate feature extraction. Deploying detection models on embedded devices presents a significant challenge due to their constrained computational resources. Unfortunately, the computing power of the NVIDIA Jetson TX2 is only 1/882nd of that of the NVIDIA H100, highlighting the embedded devices’ inability to handle such demanding calculations within a reasonable timeframe.

In the context of deployment on agricultural inspection robots and resource-constrained devices, while some detection networks boast high accuracy, their extensive parameters and computations strain devices. Conversely, the most lightweight detection models offer faster detection but often sacrifice accuracy, posing challenges for application. Thus, ensuring the model lightweight while maintaining detection accuracy is a fundamental requirement for deploying the cotton disease detection model on agricultural inspection robots and other resource-constrained devices. The CDDLite-YOLO model adeptly amalgamates the strengths of various lightweight modules such as C2f-Faster, Slim-neck, and PCDetect. By doing so, it achieves a harmonious balance between enhancing accuracy and streamlining model efficiency, rendering it well-suited for deployment on agricultural inspection robots and other resource-constrained agricultural devices.

### 4.2 Discussions of the detection results

Extensive research has been conducted on detecting cotton diseases using deep learning. However, previous studies, such as those by (Priya et al., 2021; Devi Priya et al., 2022; Susa et al., 2022; Zhang et al., 2022; Zhang et al., 2023b, Zhang et al., 2023c), did not fully consider the requirement for fast detection in applications involving agricultural inspection robots or detection conducted within controlled environments. This study addresses these specific needs.

The advantages of the CDDLite-YOLO model are as follows:

(1) Lightweight and Speed: The CDDLite-YOLO model exhibits lightweight characteristics and reduces model size, making it well-suited for deployment on agricultural inspection robots and other resource-constrained agricultural devices.
(2) Balance of Accuracy and Efficiency: The CDDLite-YOLO model strikes a harmonious balance between detection speed, accuracy, and model size, positioning it as a promising candidate for deployment on an embedded GPU chip without compromising performance.

### 4.3 Limitations and future prospects

While our proposed method has demonstrated encouraging results, there are still certain limitations that need to be addressed in future research.

The mAP@0.5 of the CDDLite-YOLO model for detecting cotton verticillium wilt diseases currently stands at 78.1%, leaving room for improvement. This lower accuracy may be attributed to factors such as background interference, as the color of cotton verticillium wilt diseases closely resembles that of the soil, making them easily blend into the background. Additionally, cotton verticillium wilt diseases and cotton Fusarium wilt diseases share a similar color, leading to occasional misdetections. To address these limitations, future experiments will explore the use of spectral imaging or hyperspectral imaging to capture more detailed information about the spectral characteristics of cotton verticillium wilt diseases. This can aid in distinguishing them from the soil background. Moreover, we will enrich our dataset by gathering and analyzing images of cotton diseases from various varieties and regions captured by agricultural inspection robots during their operation. This initiative will further validate the applicability of the model proposed in this study. Furthermore, we intend to implement systems that integrate human expertise to validate and refine model predictions, thus strengthening the accuracy of disease detection.

Regarding model deployment, we have successfully deployed the CDDLite-YOLO model on embedded devices such as the NVIDIA Jetson AGX Orin, NVIDIA Jetson TX2, and NVIDIA Jetson Nano. It performs well and fulfills the requirements for low computational power embedded devices in detecting cotton diseases in natural field environments. It achieves a balance between detection speed, accuracy, and model size, allowing deployment on these embedded GPU chips without sacrificing performance. Additionally, the CDDLite-YOLO model has been applied on agricultural inspection robots equipped with NVIDIA Jetson AGX Orin, demonstrating excellent performance in rapidly inspecting. We hope to deploy it on more cost-effective agricultural inspection robots in the future. However, our lab currently lacks access to agricultural inspection robots which are equipped with more cost-effective devices like NVIDIA Jetson Nano, which will be the focus of our future research.

Despite its limitations, CDDLite-YOLO serves as a valuable technical reference for detecting cotton diseases in natural field conditions. The application of the CDDLite-YOLO model in agricultural inspection robots for cotton disease detection holds the promise of validating its reliability.

## 5 Conclusions

Cotton, a crucial global source of natural textile fibers, is highly susceptible to cotton diseases, which significantly impact both cotton quality and yield. The use of deep learning has become an integral approach to cotton disease detection. However, current detection models often suffer from an overabundance of model parameters, making them unsuitable for resource-constrained devices and hindering the delicate balance between detection accuracy and speed. To address these challenges, our research establishes a dedicated dataset for cotton disease detection. Building upon the YOLOv8 model, we introduce significant improvements, resulting in the CDDLite-YOLO model that meets the demands for accuracy, lightweight design, and real-time performance in agricultural inspection robots and resource-constrained agricultural devices. These enhancements encompass the introduction of the C2f-Faster module, Slim-neck structure, the PCDetect detection head, and the MPDIoU loss function. These innovations enable automatic cotton disease detection in natural environments, even on resource-constrained agricultural devices. Our experimental results validate the model’s effectiveness, achieving an impressive mAP@0.5 of 90.6%. It outperforms comparable models in mAP@50–95, precision, and recall. The model excels in computational efficiency, with parameters totaling 1.8M, FLOPS at 3.6G, and a rapid detection speed of 222.22ms. These advancements represent a significant leap compared to mainstream lightweight detection models like YOLOv5n, YOLOv6n, YOLOv7-tiny, and YOLOv8n, rendering them highly suitable for deployment on agricultural inspection robots. This study provides innovative methods for developing lightweight cotton disease detection models and deploying them on agricultural inspection robots and other resource-constrained agricultural devices. Additionally, it is also a reference for crop loss estimation, pesticidal management practices, and understanding symptom-environment relationships. the CDDLite-YOLO model for detecting cotton verticillium wilt indicates room for improvement. This limitation could potentially be addressed by exploring the use of spectral imaging or hyperspectral imaging to capture more detailed information about the spectral characteristics of cotton verticillium wilt diseases.

## Data availability statement

The raw data supporting the conclusions of this article will be made available by the authors, without undue reservation.

## Author contributions

PP: Conceptualization, Methodology, Writing – original draft, Writing – review & editing. MS: Data curation, Software, Writing – review & editing. PH: Data curation, Writing – review & editing. LiH: Funding acquisition, Supervision, Writing – review & editing. SZ: Validation, Writing – review & editing. LoH: Formal analysis, Writing – review & editing. GZ: Funding acquisition, Project administration, Writing – review & editing. JZ: Funding acquisition, Project administration, Writing – review & editing.
